# LOOSE COUPLING? A CAREGIVER, A GUARDIAN, AND AN ABUSER JUMP FROM A PLANE WITH ONE PARACHUTE: WHO SURVIVES?

**DOI:** 10.1093/geroni/igae098.1857

**Published:** 2024-12-31

**Authors:** Kathleen Wilber

**Affiliations:** University of Southern California, Los Angeles, California, United States

## Abstract

My career in aging has given me the privilege of working directly with older people, training students in gerontology, partnering with remarkable leaders, and conducting studies on key questions needing evidence-informed answers. Over the half century that I have been doing applied research, healthy aging and longevity have emerged as fundamental achievements and key cultural and political priorities. In this lecture, I discuss intervention evidence and the need for research intersectionality in interventions that support family caregivers; preventing and addressing elder mistreatment; long term services and supports; and the role of supportive age-friendly communities in healthy aging. The lecture emphasizes underlying values including balancing doing too much and doing too little for older adults who need care, including those living with dementia; promoting person and family centered approaches; and the important role of diversity, equity, and inclusion in advancing innovative interventions that promote high quality long lives for all.

